# B cell-derived transforming growth factor-β1 expression limits the induction phase of autoimmune neuroinflammation

**DOI:** 10.1038/srep34594

**Published:** 2016-10-06

**Authors:** Kristbjörg Bjarnadóttir, Mahdia Benkhoucha, Doron Merkler, Martin S. Weber, Natalie L. Payne, Claude C. A. Bernard, Nicolas Molnarfi, Patrice H. Lalive

**Affiliations:** 1 Department of Pathology and Immunology, Faculty of Medicine, University of Geneva, Geneva, Switzerland; 2 Division of Clinical Pathology, Geneva University Hospital, Geneva, Switzerland; 3 Department of Neuropathology, University Medical Center, Georg August University, Göttingen, Germany; 4 Department of Neurology, University Medical Center, Georg August University, Göttingen, Germany; 5 Australian Regenerative Medicine Institute, Monash University, Clayton, Victoria, Australia; 6 Department of Neurosciences, Division of Neurology, University Hospital of Geneva and Faculty of Medicine, Geneva, Switzerland

## Abstract

Studies in experimental autoimmune encephalomyelitis (EAE), a murine model of multiple sclerosis (MS), have shown that regulatory B cells modulate the course of the disease via the production of suppressive cytokines. While data indicate a role for transforming growth factor (TGF)-β1 expression in regulatory B cell functions, this mechanism has not yet been tested in autoimmune neuroinflammation. Transgenic mice deficient for TGF-β1 expression in B cells (B–TGF-β1^−/−^) were tested in EAE induced by recombinant mouse myelin oligodendrocyte glycoprotein (rmMOG). In this model, B–TGF-β1^−/−^ mice showed an earlier onset of neurologic impairment compared to their littermate controls. Exacerbated EAE susceptibility in B–TGF-β1^−/−^ mice was associated with augmented CNS T helper (Th)1/17 responses. Moreover, selective B cell TGF-β1–deficiency increased the frequencies and activation of myeloid dendritic cells, potent professional antigen-presenting cells (APCs), suggesting that B cell-derived TGF-β1 can constrain Th1/17 responses through inhibition of APC activity. Collectively our data suggest that B cells can down-regulate the function of APCs, and in turn encephalitogenic Th1/17 responses, via TGF-β1, findings that may be relevant to B cell-targeted therapies.

The demonstration that B cell depletion by anti-CD20 monoclonal antibodies (mAbs) can lead to significant benefit to patients with multiple sclerosis (MS) has provided strong evidence of B cell involvement in MS pathogenesis^1^,^2^. Pathogenic autoreactive B cells, independent from their differentiation into Ab-secreting plasma cells^3^,^4^,^5^, can aggravate central nervous system (CNS) inflammation by contributing to the generation or reactivation of robust CNS-directed CD4^+^ T cell responses. Several lines of evidence suggest that B cells in MS may be inherently polarized toward a functional proinflammatory phenotype^6^,^7^ and that peripheral antigen (Ag)-driven B cell activation can lead to CNS autoimmune reactions^8^,^9^. However, not all B cells in MS patients harbor pathogenic potential as some evidence from patients indicate a protective role for regulatory B cells in MS. Augmented frequencies of regulatory B cells have been reported in MS patients^10^,^11^, as have defects in regulatory B cell functions^6^,^12^,^13^. While exacerbation of MS activity as a result of anti-CD20-mediated B cell depletion has not yet been reported, increased proinflammatory monocytic activity was reported in experimental autoimmune encephalomyelitis (EAE)^14^, a model for MS, and more recently in some anti-CD20 mAbs-treated MS patients^15^. These cautionary data emphasize that B cell depletion can be deleterious in some situations, and therefore supports further development of this therapeutic option for treating MS patients that spares regulatory B cell functions^16^.

Similar to the immune suppressor feature of regulatory T cells (Treg), the production of potent immunoregulatory cytokines has been noted in regulatory B cells. While the protective function of B cells in EAE and other disease models has primarily been associated with interleukin (IL)-10^17^,^18^,^19^, mouse B cells can inhibit immunity independently of IL-10^20^. Similar to mice lacking IL-10 production by B cells^21^, mice in which only B cells and B cell-derived plasma cells did not express IL-35 were shown to lose their ability to recover from EAE^22^. Despite the recognized importance of TGF-β1 in controlling the immune system^23^, no work to date has distinctly linked the *in vivo* regulatory functions of B cells to the production of TGF-β1. Tian and colleagues (2001) were the first to report that transfusion of activated B cells secreting anti-inflammatory TGF-β could impair the activity of antigen presenting cells (APCs) and inhibit Th1 responses and in turn insulin dependent diabetes mellitus^24^. Cell surface-associated TGF-β1 on activated murine B cells was later shown by Parekh and colleagues (2003) to exert potent *in vitro* inhibitory effects on CD8^+^ T cells^25^. In recent years, several *in vitro* assays or experimental models whereby cells were adoptively transferred have further revealed that B cell subpopulations expressing TGF-β can control Treg induction, immune tolerance promotion, and/or innate and adaptive immune response suppression^26^,^27^,^28^,^29^,^30^,^31^,^32^,^33^,^34^,^35^,^36^,^37^,^38^. While these studies altogether support a role for TGF-β in the regulatory capacity of B cells, the direct demonstration that TGF-β1–producing regulatory B cells modulate the immune system *in vivo* is lacking. The need of an *in vivo* demonstration is further supported by data indicating that while the three TGF-β (TGF-β1 - TGF-β2 - TGF-β3) isoforms identified to date have similar *in vitro* properties they exert discrete non-overlapping functions *in vivo*^23^.

Here, to evaluate the importance of B cell-derived production of TGF-β1 in autoimmune neuroinflammation, we engineered mice selectively deficient in TGF-β1 expression in B cells (B–TGF-β1^−/−^ mice) and tested them in EAE induced by recombinant mouse MOG (rmMOG) protein, an EAE setting in which B cells contribute functionally to the disease process. Compared to littermate controls, transgenic mice with B cell-specific deletion of TGF-β1 developed earlier onset of EAE and higher cumulative disease burden, and exhibited enhanced production of GM-CSF by Th17 cells as well as IFN-γ by Th1 cells in the CNS. No differences in regulatory T cell (Treg) levels were observed. Moreover B–TGF-β1^−/−^ mice expressed a higher frequency of CD11c^+^CD11b^+^ conventional myeloid splenic dendritic cells (DCs), as well as increased surface expression levels of MHC class II and CD86 molecules by those cells. This suggests that TGF-β1–producing B cells restrained disease progression, at least partly, by affecting the immunogenic functions of myeloid DCs. Collectively, these data show that TGF-β1 production by B cells is an important regulatory mechanism in T cell-mediated autoimmune diseases such as EAE and MS.

## Results

### TGF-β1–producing regulatory B cells limit the induction phase of EAE

**Figure 1:**
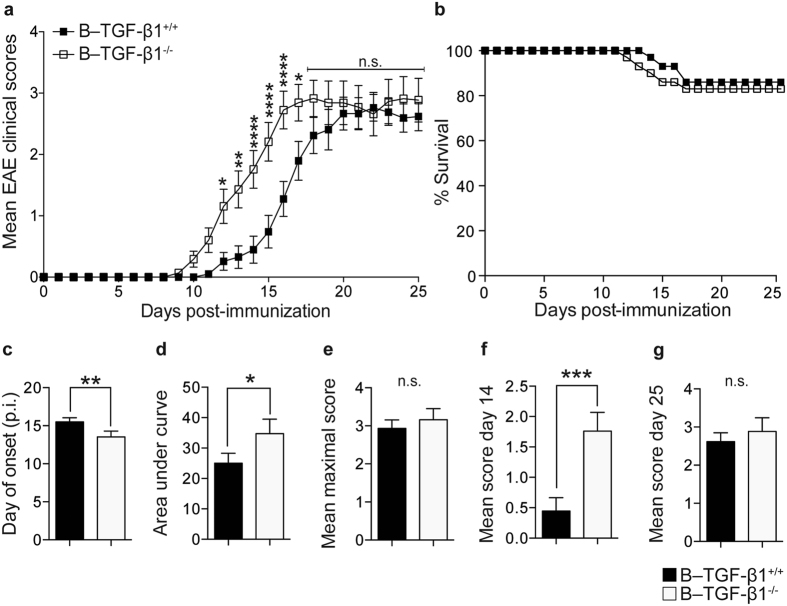
B cell-derived TGF-β1 delays the induction phase of EAE induced by mouse MOG protein. (**a**) B–TGF-β1^+/+^ (black) and B–TGF-β1^−/−^ (open) mice were immunized with recombinant mouse MOG (rmMOG) protein and monitored daily for neurologic signs. Results are presented as mean ± SEM for each day post-immunization. Two-way ANOVA was used to compare the daily EAE scores (*P < 0.05; **P < 0.01; ***P < 0.001; and ****P < 0.0001). (**b**) Percentage survival of mice during EAE course. (**c**) Day of EAE onset, (**d**) area under the curve (AUC), (**e**) mean maximal clinical score, (**f**) mean maximal score during acute phase (day 14), (**g**) mean maximal score during chronic phase (day 25). Data are mean ± SEM (*P < 0.05; **P < 0.01; and ***P < 0.001 by Mann-Whitney U-test. Results are a composite of 3 independent experiments of n = 9–10 mice/group with similar results.

The role of TGF-β1 production by B cells was investigated in EAE induced by rmMOG protein, a protocol that activates the cellular component of B cell immunity^14^. Mice containing TGF-β1–deficient B cells (B–TGF-β1^−/−^) and control (B–TGF-β1^+/+^) mice were monitored for up to 25 days post-immunization (chronic phase). There was a significant difference between B–TGF-β1^+/+^ and B–TGF-β1^−/−^ mice, as shown by the comparison of the mean EAE scores monitored over a period of 25 days (Fig. 1a). No difference in survival was found between groups over the 25 days of disease (Fig. 1b). Selective B cell TGF-β1–deficiency caused an earlier onset and peak disease (Fig. 1c). Moreover, an increase of the area under curve (AUC) (equivalent of the cumulative total EAE scores) was observed in absence of TGF-β1–producing B cells (Fig. 1d). Despite faster development of the clinical disease in B–TGF-β1^−/−^ mice, both sets of mice eventually reached similar EAE severity, as measured by the comparison of maximum EAE scores (Fig. 1e). Of note, B–TGF-β1^−/−^ mice showed higher EAE scores during the acute (day 14) (Fig. 1f), but not the chronic (day 25) (Fig. 1g) phase of the disease. These data suggest that TGF-β1–producing B cells are maximally effective during early EAE initiation, while they may not have a direct role during disease progression.

### B cell-specific TGF-β1 deficiency increases CNS inflammation

**Figure 2:**
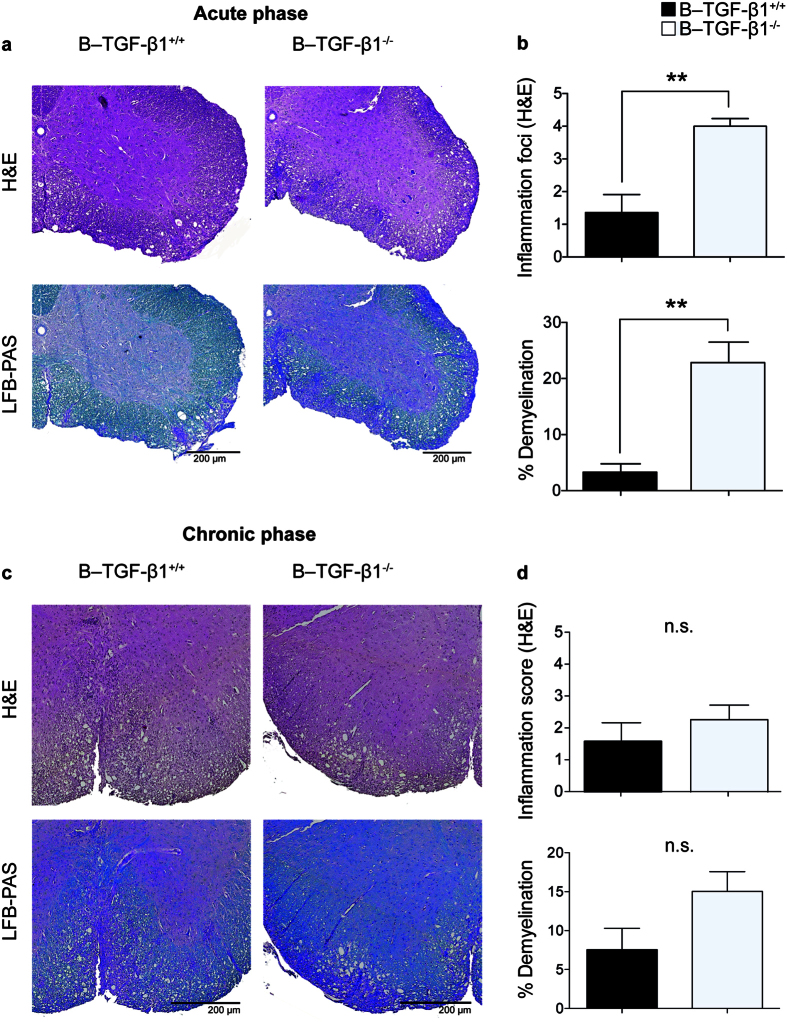
EAE acceleration in B–TGF-β1^−/−^ mice is associated with augmented spinal cord inflammation and demyelination. Mice selected for histologic examination had clinical scores at the time of sacrifice that represented the mean for their group. (**a**) Representative images of paraffin-embedded spinal cord sections from indicated groups sacrificed on day 14 stained for H&E or Luxol Fast Blue/PAS stain (LFB-PAS) are shown. The average number of inflammatory lesions (**b**, top) and extent of demyelination (**b**, bottom) is shown. (**c**) Representative images of spinal cord sections from indicated groups sacrificed on day 25 stained for H&E or LFB-PAS are shown. The average number of inflammatory lesions (**d**, top) and extent of demyelination (**d**, bottom) is shown. Bar = 200 μM, 5–8 sections of spinal cord per mouse (n = 5 per group) are presented as bar graphs, mean ± SEM. **P <  0.01 by Student’s *t*-test.

Histological features of EAE induced by active sensitization with rmMOG protein are inflammation and demyelination. To assess the degree of inflammation and demyelination during the acute (day 14) and chronic (day 25) phase of the disease, spinal cord (SC) sections from EAE mice were stained with hematoxylin and eosin (H&E) or luxol fast blue/PAS (LFB-PAS), respectively. Histological examination of SC disease burden corroborated the clinical EAE score assessments during the acute and chronic phases of the disease: as shown in Fig. 2a,b, the extent of inflammation and demyelination was higher in B–TGF-β1^−/−^ mice than in B–TGF-β1^+/+^ mice during acute EAE. During the chronic phase, inflammatory cell infiltration and demyelination were similar between the groups of mice (Fig. 2c,d). Taken together, these data show that selective ablation of TGF-β1–producing B cells increased disease susceptibility and neuroinflammation during acute EAE disease.

### Increased disease severity in B–TGF-β1^−/−^ mice is associated with enhanced frequencies of proinflammatory T cells in the CNS

**Figure 3:**
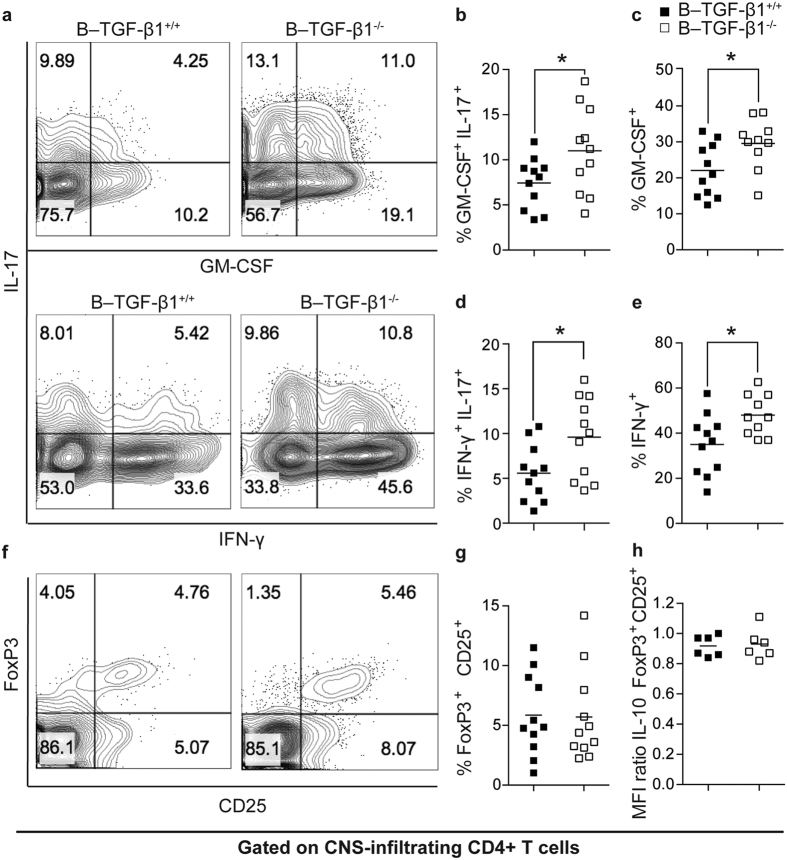
Increased EAE susceptibility in B–TGF-β1^−/−^ mice correlates with augmented CNS infiltration of proinflammatory Th1 and Th17 cells. (**a–e**) Cytokine production by CD4^+^ T lymphocytes isolated on day 14 after disease induction from the spinal cords (SCs) of B–TGF-β1^+/+^ and B–TGF-β1^−/−^ EAE-affected mice. The cells were stimulated *in vitro* with PMA/Ionomycin for 4 hrs and incubated with Brefeldin A and stained for extracellular markers and intracellular cytokines. Representative FACS plots (gated on CD4^+^ cells) from mice from each group are shown in (**a**). Quadrant gates were set using a FMO sample. (**b–e**) Tabulated results are presented as percentage of CD4^+^ T cells in the SC infiltrate. (**f,g**) The percentage of CD4^+^CD25^+^FoxP3^+^ Tregs was evaluated in the SCs on day 14 after disease induction by intracellular staining. Representative contour plots (**f**) are shown including quantification (**g**). Quadrant gates were set using a FMO sample. (**h**)Intracellular IL-10 production in CD4^+^CD25^+^FoxP3^+^ Tregs. Results are reported as relative (ratio) geometric mean of fluorescence intensity (MFI) value. Results are presented as mean ± SEM (n = 11) (*P < 0.05 by Student’s *t*-test). Results are a composite of 2 independent experiments with comparable results.

We next examined the potential effects of B cell-specific TGF-β1 deficiency in rmMOG protein-induced EAE on proinflammatory CNS T cell responses. To represent the acute inflammatory phase of EAE disease, we chose day 14 post-induction. Consistent with clinical and histological observations, the overall numbers (data not shown) and frequencies of CNS-infiltrating Th1 and Th17 cells during the acute stage of the disease were more extensive in B–TGF-β1^−/−^ mice when compared to B–TGF-β1^+/+^ mice (Fig. 3a). While CNS-infiltrating CD4^+^ T cells showed equivalent production of IL-17A in both groups (data not shown), a higher population of CD4^+^ T cells that expressed both IL-17A and GM-CSF, a well-known Th17 cytokine, was found in B–TGF-β1^−/−^ mice in comparison to B–TGF-β1^+/+^ mice (Fig. 3b). CNS CD4^+^ T cells from B–TGF-β1^−/−^ mice also globally showed increased production of GM-CSF (Fig. 3c). As for the Th1–type cytokine, a larger population of CNS-infiltrating CD4^+^ T cells expressing IFN-γ along with IL-17A was detected in B–TGF-β1^−/−^ mice in comparison to B–TGF-β1^+/+^ mice (Fig. 3d). Likewise, total IFN-γ production by CNS CD4^+^ T cells was augmented in B–TGF-β1^−/−^ mice in comparison to B–TGF-β1^+/+^ mice (Fig. 3e). Interestingly, no differences in the levels of CNS-infiltrating CD4^+^CD25^+^FoxP3^+^ regulatory T cells (Tregs) were noted between the groups of mice (Fig. 3f,g), nor was there any difference in the ability of Tregs to produce IL-10 (Fig. 3h).

### Selective B cell TGF-β1–deficiency increases peripheral proinflammatory Th17 responses

**Figure 4:**
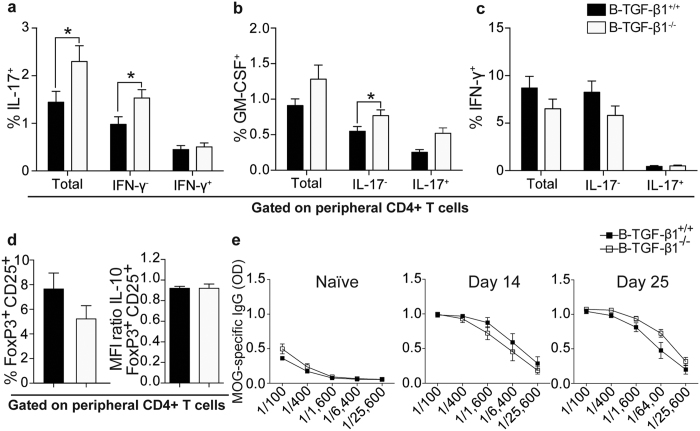
B cell-derived TGF-β1 expression restrains the *in vivo* development of Th17 responses. (**a–e**) Splenocytes from B–TGF-B1^+/+^ and B–TGF-B1^−/−^ EAE mice collected at the acute phase of the disease were stimulated *in vitro* with PMA/Ionomycin for 4 hrs and incubated with Brefeldin A and stained for CD3, CD4, CD25, FoxP3 and intracellular cytokines. Tabulated results from all mice are presented as percentage of live CD3^+^CD4^+^ T cells in the spleen. (**a**) Total: all IL-17^+^ cells; IFN-γ^−^: IL-17^+^IFN-γ^−^ cells; IFN-γ^+^: IL-17^+^IFN-γ^+^ cells. (**b**) Total: all GM-CSF^+^ cells; IL-17^−^: GM-CSF^+^IL-17^−^ cells; IL-17^+^: GM-CSF^+^IL-17^+^ cells. (**c**) Total: all IFN-γ^+^ cells; IL-17^−^: IFN-γ^+^IL-17^−^ cells; IL-17^+^: IFN-γ^+^IL-17^+^ cells. (**d**, left) Frequencies of CD4^+^CD25^+^FoxP3^+^ Tregs. (**d**, right) Quantification of intracellular IL-10 production in Tregs. Results are reported as relative (ratio) geometric MFI value. Results are presented as mean ± SEM (n = 11) (*P < 0.05 by Student’s *t*-test). Results are a composite of 2 independent experiments with comparable results. (**e**) Sera from individual naive and immunized (rmMOG) B–TGF-B1^+/+^ and B–TGF-B1^−/−^ mice were obtained on days 14 and 25 after immunization, and serum MOG-specific IgG levels were calculated. MOG-specific IgG titers are expressed as mean OD values ± SEM from one of three representative experiments (performed in triplicate) from 1:4 serial dilutions.

As EAE was accelerated in B–TGF-β1^−/−^ mice, suggesting a critical role for TGF-β1–producing B cells in the priming of T cells during EAE, we next evaluated how deficiency of TGF-β1 expression by B cells influenced the peripheral development of pathogenic cells *in vivo*. Compared with control mice, B–TGF-β1^−/−^ demonstrated normal spleen cellularity (data not shown). As shown by intracellular flow cytometry during acute EAE (day 14), levels of splenic CD4^+^ T cells expressing IL-17A was increased in B–TGF-β1^−/−^ mice in comparison to B–TGF-β1^+/+^ mice (Fig. 4a). Moreover, a larger proportion of splenic CD4^+^ T cells that produce GM-CSF was detected in B–TGF-β1^−/−^ mice than in B–TGF-β1^+/+^ mice (Fig. 4b). Peripheral IFN-γ–producing CD4^+^ T cells were found in similar frequencies in both groups (Fig. 4c). Finally, comparable percentages of CD25^+^FoxP3^+^CD4^+^ Tregs and intracellular expression levels of IL-10 by these cells were also noted (Fig. 4d), discounting a function for B cell-derived TGF-β1 expression in driving the generation/expansion or survival of Tregs *in vivo*. Furthermore, as recent data indicate that MOG-specific Abs may accelerate CNS inflammation and disease by an interplay with T cells^39^,^40^ and that B-cell derived TGF-β1 can control T-dependent responses^41^, we evaluated whether selective ablation of TGF-β1 production in B cells was associated with increased titers of MOG-reactive IgGs. Similar serum levels of MOG-specific IgGs were detected in both B–TGF-β1^+/+^ and B–TGF-β1^−/−^ mice prior to disease induction as well as during the acute and chronic phases of EAE (Fig. 4e). These data suggest that the more pronounced proinflammatory T cell phenotype and accelerated EAE seen B–TGF-β1^−/−^ mice does not implicate altered auto-Ab production.

### B cell TGF-β1–deficiency augments the frequencies of myeloid DCs and activation status of antigen APCs

**Figure 5:**
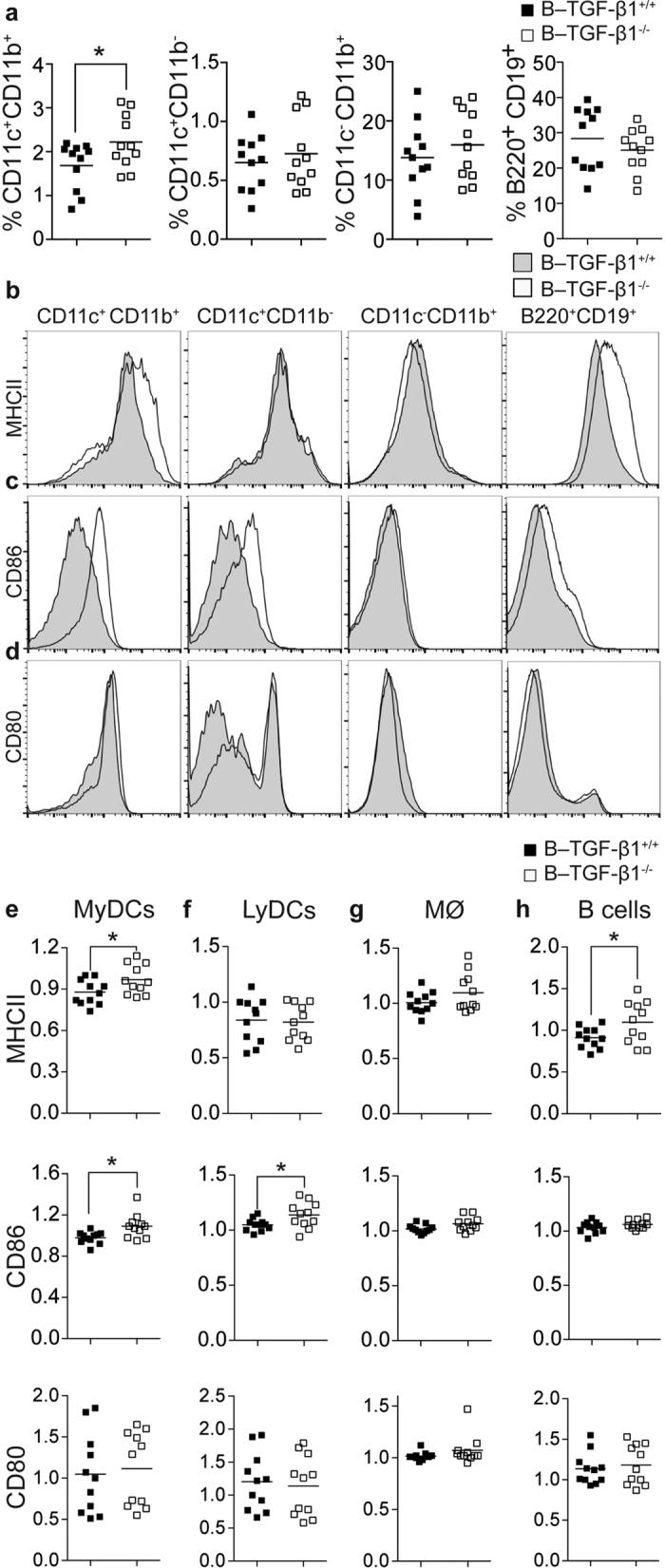
Selective B cell TGF-β1–deficiency increases the frequencies and immunogenicity of APCs. B–TGF-β1^+/+^ (black) and B–TGF-β1^−/−^ (open) mice were immunized with rmMOG protein and APC subpopulations were characterized by flow cytometry during the acute phase of the disease (day 14). (**a**) Frequency of myeloid (CD11c^+^CD11b^+^) DCs, lymphoid (CD11c^+^CD11b^−^) DCs, monocytes/macrophages (CD11c^−^CD11b^+^), and B cells (B220^+^CD19^+^). Surface expression of (**b**) MHC class II, (**c**) CD86, and (**d**) CD80 was measured in each cell subpopulation and presented as mean fluorescence (MFI). Representative histograms are shown. (**e–h**) Tabulated results are reported as relative (ratio) geometric MFI values for (**e**) myeloid DCs (MyDCs) (**f**) lymphoid DCs (LyDCs), (**g**) monocytes/macrophages (MØ), and (**h**) B cells. Data are a composite of two independent experiments with similar results; *P < 0.05 by Student’s *t*-test.

Data from our B–TGF-β1^−/−^ mice indicate that B cell-derived TGF-β1 production restrains the development of proinflammatory T cell subsets. As B cells can exert anti-inflammatory properties via inhibiting the maturation and proinflammatory differentiation of other APCs *in vivo*^14^,^42^, we evaluated the influence of selective B cell TGF-β1–deficiency on the frequency of specific populations of APCs during the acute phase of EAE. Selective B cell TGF-β1–deficiency was associated with a higher frequency of CD11c^+^CD11b^+^ myeloid DCs in EAE mice (Fig. 5a), suggesting that B cell-derived TGF-β1 expression could control the turnover of this APC subset. In contrast to myeloid DCs, no differences were observed in the frequencies of splenic CD11c^+^CD11b^−^ lymphoid DCs, CD11c^−^CD11b^+^ monocytes/macrophages, or B220^+^CD19^+^ B cells (Fig. 5a). Interestingly, no changes between groups were found in naïve mice, suggesting that B cell-derived TGF-β1 expression specifically control the development of myeloid DCs during inflammation (Supplementary Fig. S1). We next evaluated whether selective B cell TGF-β1–deficiency caused aberrant expression of MHC class II (Fig. 5b), and co-stimulatory CD86 (Fig. 5c) and CD80 (Fig. 5d) molecules. As shown in Fig. 5e, CD11c^+^CD11b^+^ myeloid DCs expressed higher levels of MHC class II and CD86 in B–TGF-β1^−/−^ mice than in B–TGF-β1^+/+^ mice. No differences were however observed for CD80 expression (Fig. 5e). While increasing CD86 expression by lymphoid DCs (Fig. 5f), selective B cell TGF-β1–deficiency had no effect on monocyte/macrophage expression of MHC class II, CD86 or CD80 (Fig. 5g). Finally, TGF-β1–deficient B cells had elevated surface levels of MHC class II molecules (Fig. 5h). Altogether, these data suggest that lack of TGF-β1–producing regulatory B cells favors the activation of inflammatory T cells and the development of more activated APCs.

### TGF-β1–deficient B cells promote normal proinflammatory CD4^+^ T cell responses

**Figure 6:**
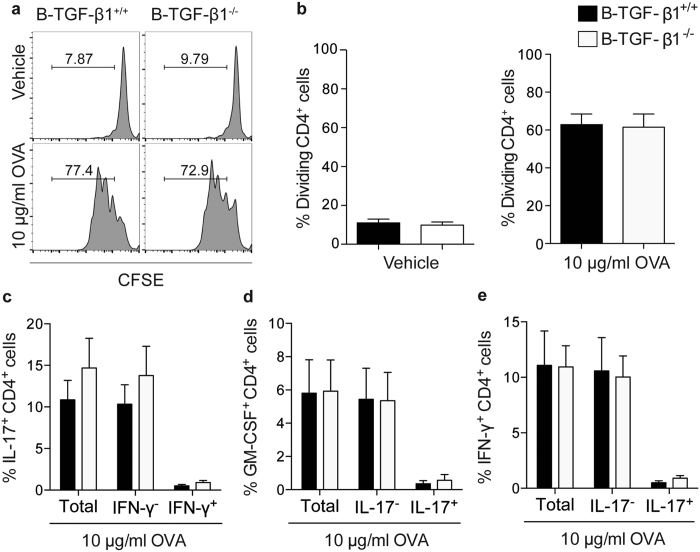
TGF-β1 produced by B cells does not directly affect proinflammatory T cell activation. MACS-separated B cells (purity >95%) isolated from OVA-immunized mice were co-cultured with naïve T cells isolated from OVA-specific T cell receptor Tg mice in the presence of endotoxin-free OVA protein. (**a,b**) T cell proliferation was evaluated by dilution of CFSE fluorescence intensity. Representative FACS plots (gated on CD4^+^ cells) from mice from each group are shown in (**a**). Tabulated results are presented in (**b**) as percentage of proliferative CD4^+^ T cells. (**c–e**) Proinflammatory T cell differentiation was evaluated by secretion of (**c**) IL-17, (**d**) GM-CSF, and (**e**) IFN-γ, as described in Fig. 4. Data are a composite of two independent experiments (n = 3 mice/group) with similar results.

B cells are essential for generating optimal pathogenic CD4^+^ T cell responses following recombinant protein immunization^3^,^14^. As B cells from B–TGF-β1^−/−^ EAE mice showed increased levels of MHC class II surface expression (Fig. 5h) we next addressed whether TGF-β1–deficient B cells from EAE mice had increased capacity to promote the activation of CD4^+^ T cells. To study B cell APC function in the absence of any possible effects caused by residual endotoxin contamination commonly found in recombinant protein preparations, B cells were collected from B–TGF-β1^+/+^ and B–TGF-β1^−/−^ mice immunized with OVA protein and used as APCs in co-cultures with OVA-specific TCR Tg cells (OT-II cells) and endotoxin-free OVA protein. Immunization of mice with intact OVA protein was previously shown to be as potent as immunization with rmMOG to activate B cells *in situ*, suggesting that B cell activation is a characteristic associated with immunization with protein^14^. Despite increased expression of MHC class II molecules, B cells from B–TGF-β1^−/−^ mice showed no superior capacity to induce T cell proliferation in comparison to B cells from B–TGF-β1^+/+^ in response to OVA protein (Fig. 6a,b). Further analysis of proinflammatory cytokine production by CD4^+^ T cells showed no differences in their expression of IL-17 (Fig. 6c), GM-CSF (Fig. 6d) or IFN-γ (Fig. 6e). Altogether, these data indicate that selective B cell TGF-β1–deficiency does not enhance APC function of activated B cells and that augmented proinflammatory T cell responses in B–TGF-β1^−/−^ EAE mice likely reflect increased APC function of more professional APCs such as myDCs.

### Selective B cell TGF-β1–deficiency augments the capability of residual APCs to activate proinflammatory T cells

**Figure 7:**
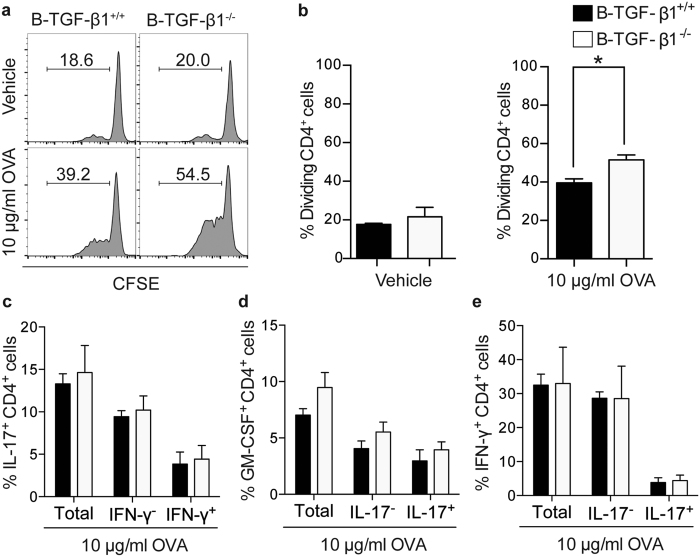
Selective B cell TGF-β1–deficiency increases the capacity of remaining APCs to generate proinflammatory T cells. Remaining splenocytes (after *in vitro* depletion of B and T lymphocytes) isolated from OVA-immunized mice were co-cultured with naïve T cells isolated from OVA-specific T cell receptor Tg mice in the presence of endotoxin-free OVA protein. (**a,b**) T cell proliferation was evaluated by dilution of CFSE fluorescence intensity. Representative FACS plots (gated on CD4^+^ cells) from mice from each group are shown in (**a**). Tabulated results are presented in (**b**) as percentage of proliferative CD4^+^ T cells. (**c–e**) Proinflammatory T cell differentiation was evaluated by secretion of (**c**) IL-17, (**d**) GM-CSF, and (**e**) IFN-γ, as described in Fig. 4. Data are a composite of two independent experiments (n = 3 mice/group) with similar results.

Previous studies have indicated that cytokine-producing B cells may locally regulate other APCs^42^,^43^. Thus, we evaluated whether lack of TGF-β1 production by B cells influenced the function of remaining APCs. For this purpose, we isolated spleen cells from either B–TGF-β1^+/+^ or B–TGF-β1^−/−^ mice primed with OVA protein, and cultured them, after depletion of both B and T lymphocytes, with naïve OVA-specific OT-II CD4^+^ T cells and endotoxin-free OVA protein. When compared to remaining splenocytes from B–TGF-β1^+/+^ mice, splenocytes remaining from B–TGF-β1^−/−^ mice exhibited an increased capacity to promote T cell proliferation in response to OVA protein (Fig. 7a,b). While we found an increased percentage of dividing CD4^+^ T cells in co-cultures with remaining splenocytes from B–TGF-β1^−/−^ mice, no statistically significant differences were found in the expression levels of proinflammatory cytokines produced by those CD4^+^ T cells in this assay (Fig. 7c–e). In summary, these results suggest that selective deficiency of TGF-β1-producing B cells could augment the T-cell stimulatory functions of APCs *in vitro*, and therefore the Ag-specific proinflammatory T responses.

## Discussion

Evidence suggests that some B cell subsets may have an important role in immune regulation of autoimmune neuroinflammation^19^,^21^,^22^,^44^,^45^,^46^. The goal of this investigation was to evaluate the contribution of B cell-derived TGF-β1, a regulatory cytokine with pleiotropic functions in control of T cell responses, in the context of CNS autoimmunity. To this end, we created mice with TGF-β1–deficiency restricted to B cells (B–TGF-β1^−/−^ mice) and tested them in a mouse model of MS that endorses participation of B cells. In comparison to mice containing B cells that expressed TGF-β1 (B–TGF-β1^+/+^), B–TGF-β1^−/−^ mice were more susceptible to EAE induced by rmMOG and exhibited increased CNS inflammation as well as proinflammatory Th1 and Th17 responses. Our results further indicate that B cell-derived TGF-β1 production does not restrain T cell immunity by directly inhibiting proliferation or cytokine production of T cells, but rather influences the proinflammatory profile of potent professional APCs. Regulation by TGF-β1 opens up new avenues for novel populations, or mechanisms of action, of regulatory B cells in CNS autoimmunity.

Studies in various experimental animal models have identified multiple types of regulatory B cells exhibiting diverse mechanisms of immune suppression. In acute EAE models B cells were shown to orchestrate disease recovery^47^ through the production of regulatory cytokines^21^,^22^, or via interaction with Tregs^44^,^46^. While these studies indicate that regulatory B cell activities may be critical for resolving disease, additional observations also support a regulatory role for B cells during disease induction^14^,^19^,^45^. Our study indicates that regulatory TGF-β1–producing B cells exert their anti-inflammatory effects early, but not late, during the course of EAE. Mechanistically, we found that selective B cell TGF-β1–deficiency up-regulated the maturation markers CD86 and MHC class II on myeloid DCs, potent APCs that play a critical role in Ag presentation of CD4^+^ T cell activation during EAE initiation, while not affecting the generation or maintenance of regulatory T cells, which have been shown to inhibit late-phase disease^48^.

Regulatory B cells have been reported to either directly control T cell responses through cognate interactions^49^ or to locally regulate APCs via secretion of anti-inflammatory cytokines^48^. A major mediator of B cell suppression so far is IL-10. B cell-derived IL-10 was shown to limit production of IL-12 by DCs and restrain Th1 differentiation^42^,^50^. Likewise, TLR-activated B cells limit the capacity of DCs to secrete IL-6 and IL-23 and to induce Th17 differentiation via IL-10^43^. In addition to IL-10 production, TGF-β1 expression has also been shown to regulate CNS autoimmune T cell responses indirectly through DCs. More specifically, data indicate that the lack of TGF-β1 signaling in DCs is sufficient to promote severe EAE^51^. Here, our data suggest that B cell-derived TGF-β1 decreases the Ag-presenting capacity and/or co-stimulatory activity of myeloid DCs. As myeloid DCs recently emerged as a unique APC population capable of driving Th17 differentiation in CNS during EAE^52^, our data stress the importance of the interplay of TGF-β1 and DCs in the control of EAE.

In contrast to changes seen during EAE, frequency and phenotype of myeloid DCs were unaffected in naïve mice containing selective B cell TGF-β1 deficiency (Fig. S1). These observations suggest that inflammatory signals may be essential for the development of TGF-β1–producing B cells. The importance of inflammation in the development of regulatory TGF-β1–expressing B cells calls into question the location of their maturation during EAE pathogenesis. While most EAE studies characterized the spleen as the primary location for regulatory B cell development^53^, regulatory B cells in the draining lymph nodes (dLNs) of EAE mice have recently been reported to be critical in restraining autoimmune neuroinflammation by inhibition of DC function in the development of encephalitogenic T cells^54^. Whether B cell-derived TGF-β1 production exerts it suppressive function in the dLNs of EAE mice remains to be investigated. Additional mechanisms of immune suppression mediated by B cells during EAE may include accumulation within the CNS^19^. Conceivably, B cell-derived TGF-β1 could further contribute to immune modulation *in situ*. Interestingly, consistent with the regulatory function of IL-10-producing B cells involved in disease initiation, amelioration of EAE symptoms was shown to be associated to a quick expansion of these cells in the spleen but not in the CNS^48^. As our data also suggest that TGF-β1–producing B cells exert their protective functions during EAE initiation, it is unlikely that they would be contributing to regulation of CNS autoimmunity *in situ*.

Major unanswered questions are what mechanisms induce the production of TGF-β1 by cells and what subsets of B cells are exerting the TGF-β1 regulation. Recent observations established that different inflammatory environments induce distinct regulatory B cell populations^20^. Remarkably, B cells were shown to produce relatively low levels of pro- and active TGF-β in physiological conditions^55^,^56^, while they were reported to express high levels of active TGF-β in response to LPS stimulation^24^,^25^. Importantly, LPS-activated B cells were documented to comparably express much higher levels of TGF-β1 than B cells stimulated with anti-Ig plus anti-CD40 Ab, a T-dependent mode of B cell activation^25^. As mentioned earlier, microbial signals may contribute to the regulatory function of B cell-derived TGF-β1. In addition to signaling via innate receptors, evidence also suggests that B cell receptor (BCR) recognition is important in induction of regulatory B cells expressing IL-10^21^,^57^,^58^. Whether the development and function of TGF-β1–producing B cells requires Ag receptor diversity and Toll-like receptor (TLR) signals function remains to be determined. The characterization of TGF-β1–producing B cells may further shed light on whether these cells are restricted to a unique subset of regulatory B cells or, alternatively, if they represent a hallmark of an inflammatory microenvironment. The quantification of TGF-β1 expression by B cells (Supplementary Fig. S2) indicates that TGF-β1–producing B cells do not belong to the CD5^+^CD1d^hi^ B10 subset^59^ or CD138^+^ plasma cells, which have been shown to be the main source of IL-10 and IL-35 during EAE^22^,^54^. The immunophenotypical characterization of TGF-β1–expressing B cells will be an important focus of future studies. Finally, while there is ample evidence to support the clinical efficacy of pan-B cell depletion, using depleting anti-CD20 mAbs, for the treatment of MS^1^,^2^, B cell depletion may also promote the occurrence of CNS inflammation in some settings^14^,^60^,^61^,^62^,^63^. It would hence be worthwhile to evaluate whether human TGF-β1–secreting B cells^32^ can contribute to the regulation of inflammatory and autoimmune diseases, including MS.

The observation that TGF-β1 production by B cells restrained proinflammatory T cell activation, and histologic and clinical manifestations of CNS autoimmunity, provides novel insight regarding B-APC cell communication in the pathogenesis of MS and the use of B cell depletion in its therapy.

## Materials and Methods

### Mice

WT C57BL/6J CD45.1, OT-II mice, TGF-β1^−/−^ (Tgfb1^tm2.1Doe^/J), and CD19cre/cre (homozygous for cre cassette, B6.129P2(C)-*Cd19*^*tm1*(*cre*)*Cgn*^/J) mice were purchased from the Jackson Laboratories (Bar Harbor). For selective deletion of floxed TGF-β1 gene in B lymphocytes, CD19*cre/cre* mice were crossed with TGF-β1^flox/flox^ mice. Litters heterozygous for the cre cassette (CD19*cre*-TGF-β1^flox/flox^) (B–TGF-β1^−/−^) and TGF-β1^flox/flox^ (B–TGF-β1^+/+^) mice were used for experiments. Animals were housed in a specific pathogen-free barrier facility at the Medical Center of Geneva, Faculty of Medicine (Geneva, Switzerland). All breeding and experimental protocols and procedures were reviewed and approved by the Institutional Animal Care and Use Committee of the Geneva University School of Medicine (protocol number: GE/107/15). Animal care and experimental procedures were carried out in accordance with the guidelines of the Institutional Animal Care and Use Committee of the Geneva University School of Medicine.

### Antigens

Recombinant mouse MOG protein 1–117 (rmMOG) was provided C.C.A. Bernard (Monash University, Clayton) and synthesized, purified and refolded as previously reported^64^. Endotoxin-free Ovalbumin (OVA) (#vac-pova-100) was purchased from Invivogen (San Diego, USA).

### Induction and assessment of EAE

C57BL/6J mice were injected subcutaneously with 75 μg rmMOG emulsified in CFA (DIFCO Laboratories) containing 200 μg heat-killed Mycobacterium tuberculosis (Mtb) H37RA (DIFCO Laboratories) on day 0. Additionally, mice received i.v. 350 ng Bordetella pertussis toxin (Sigma-Aldrich) in 0.2 ml PBS on days 0 and 2. Individual animals were observed daily and clinical scores were assessed with a 0- to 5-point scoring system, as follows: 0 = no clinical disease, 1 = loss of tail tone only, 2 = mild monoparesis or paraparesis, 3 = severe paraparesis, 4 = paraplegia and/or quadraparesis, and 5 = moribund or death. Moribund mice were given disease severity scores of 5 and euthanized.

### T cell co-culture assays

Mice were primed with OVA protein (100 μg/mouse) emulsified with CFA and B cells and/or remaining splenocytes (after *in vitro* depletion of B cells) isolated by magnetic separation from spleen after 10 days. For B cell – T cell co-culture assays, B cells were isolated from spleens by depletion from non-B cells (CD4, CD8, CD11b, CD43, CD49b, CD90.2, Ly-6C/G (Gr-1), TER119) (EasySep™, kit #19854) according to the manufacturer’s instructions. Following separation, B cells were evaluated for purity (>99%) by flow cytometry staining for B220. When necessary, cells were enriched a second time using a fresh tube to obtain >95% cell purities. For T cell co-culture assays using remaining splenocytes as APCs, spleens were isolated and B220^+^ B cells (EasySep™, kit #18954) and CD3^+^ T cells (Ebioscience, kit #8802-6840-74) were removed by positive magnetic separation. Naïve CD4^+^ T cells were isolated from spleens of OT-II mice by depletion of non-CD4^+^ T cells (negative selection, (EasySep™, kit #19852). Cells were cultured in RPMI supplemented with 2% FCS, β-mercaptoethanol, 2 mmol/L L-glutamine, 50 IU/mL penicillin, 50 μg/mL streptomycin, 1X insulin-transferrin-selenium, and 5 μM HEPES. For CFSE dilution, 2.5 × 10^5^ purified B220^+^ B cells or remaining splenocytes were co-cultured in 96-well plates with 2 × 10^4^ naïve T cells isolated from OVA TCR Tg (OT-II) mice in the presence of endotoxin-free OVA protein. After 72 hrs, T cell proliferation was assessed by CFSE dilution assay after gating on CD4^+^ T cells. FACS or ELISA evaluated T cell differentiation after 72 hrs.

### CNS cell isolation

CNS mononuclear cells were isolated from EAE mice at peak of disease after cardiac perfusion with PBS, as previously described^3^. Briefly, minced CNS (spinal cord) tissues were digested with collagenase D (2.5 mg/ml; Roche Diagnostics, Indianapolis, IN) at 37 °C for 60 min. Mononuclear cells were isolated by discontinuous Percoll gradient (70/30%) (Sigma-Aldrich) centrifugation. Lymphocytes were collected from the 30:70% interface and washed. Total cell numbers were determined by counting on a hemocytometer, and viability was assessed by trypan blue exclusion. To exclude the possibility of generating artifacts during the isolation process of CNS-infiltrating cells, the percentages rather than the “absolute” cell numbers were displayed to facilitate comparison of results.

### Flow cytometric analysis

For six-color immunofluorescence analysis, single-cell suspensions (1 × 10^6^ cells) were incubated with anti-mouse FcRIIB/FcRIIIA mAb (2.4G2) (BD Bioscience) to avoid nonspecific staining and were subsequently stained at 4 °C using predetermined optimal concentrations of mAb for 30 min. as previously described^3^. Blood erythrocytes were lysed before staining using FACS lysing solution (BD Biosciences). Antibodies to the mouse proteins anti-CD45R (B220)-PerCP-Cy5.5 (RA3-6B2), anti-CD4-PerCP-Cy5.5 (RM4-5), anti-CD3-PE-Cy7 (145-2C11) anti-CD11c-APC (N418), anti-CD11b-PE (M1/70), anti-CD44-PE (IM7), anti-CD62L-APC (MEL-14), anti-MHCII-FITC (M5/114.15.2), anti-CD86-PE-CY7 (GL1), anti-CD80-PE (B7-1) were purchased from eBioscience. Dead cells were excluded using Live/Dead Fixable Near-IR Dead Cell Stain Kit (#L10119) from Life Technologies. Positive cells were defined using a “fluorescence minus one” (FMO) sample. Samples were processed on a FACS Cyan flow cytometer (Becton Dickinson) and analyzed using FlowJo analysis software (Tree Star, Version 10.0.8r1). The relative MFI of each sample was calculated by dividing its geometric mean value by the geometric mean value of an arbitrary positive sample from the control group. This approach allows for comparison of multiple test samples within a group and between different groups.

### Intracellular cytokine staining

Lymphocytes were stimulated *in vitro* with PMA (50 ng/ml; Sigma-Aldrich) and ionomycin (1 μg/ml; Sigma-Aldrich), in the presence of brefeldin A (1 μl/ml; Sigma-Aldrich) for 4 hrs before staining. FcR’s were blocked before cell surface staining with anti-CD3-PE-Cy7 (145-2C11, eBioscience) and anti-CD4-PerCP-Cy5.5 (RM4-5, eBioscience). Dead cells were excluded with Live/Dead Fixable Near-IR Dead Cell Stain Kit from (#L10119) from Life Technologies. After staining, cells were washed, fixed, and then permeabilized using the Cytofix/Cytoperm Plus Fixation/Permeabilization Kit (BD Biosciences) according to the manufacturer’s instructions. Permeabilized cells were then stained with anti-IL-17-FITC (eBio17B7), anti-GM-CSF-PE (MP1-22E9) and anti-IFN-γ-APC (XMG1.2) (all from eBioscience). Quadrant gates were set using a FMO sample.

### FoxP3 staining

For detection of FoxP3, FcR’s were blocked followed by cell surface staining with anti-CD3-PE-Cy7 (145-2C11), anti-CD4-PerCP-Cy5.5 (GK1.5), anti-CD25-APC (PC61.5), anti-IL-10-FITC (JESS-16E3) and anti-FoxP3-PE (FJK-16s) (all from eBioscience).

### Detection of anti-MOG antibodies

Serum MOG-specific IgG antibodies were measured using a noncommercial ELISA as previously described^65^,^66^. 96-Maxisorb plates (Costar) were precoated with rmMOG (1–117) protein (10 μg/ml in PBS), blocked with 1% BSA (Sigma-Aldrich), and incubated with sera for 2 hrs at the indicated dilution. After washing, MOG-specific IgG retained by the plate-bound MOG was detected with horseradish-peroxidase–conjugated anti–mouse IgG (eBioscience). OVA protein-coated plates were used as negative controls for nonspecific binding. SOFTmax ELISA plate reader (450-nm wavelength) and software (Molecular Devices) were used for data analysis.

### Histology and immunohistochemistry

Spinal cords (SCs) were removed and fixed in 4% neutral-buffered formalin, paraffin-embedded and sectioned (3 μM). Representative sections were stained with Luxol fast blue (LFB)-hematoxylin and eosin (H&E) (for inflammation and demyelination) and examined by light microscopy and scanned with Axioscan.Z1 (Zeiss). The number of inflammatory foci per section was counted in each H&E-stained section in a blinded fashion. The degree of demyelination was determined as a percentage of demyelinated area in comparison to total area of white matter in SC sections.

### RNA Isolation and Real-time Quantitative PCR

RNA was prepared from MACS-sorted CD19^+^ B cells or FACS-sorted CD138^−^, CD138^+^ plasma cells, CD19^+^CD5^−^ CD1d^lo^ or CD19^+^CD5^+^CD1d^hi^ Bregs using Qiagen RNAeasy Mini Kits and subjected to DNase I (Roche Diagnostics) digestion. Random hexamer primers (Promega, Madison, WI) and Superscript II RNase H reverse transcriptase (Invitrogen, Carlsbad, CA) were used to generate cDNA. TGF-β1 transcripts were quantified by real-time PCR analysis using SYBR Green as the detection agent. The PCR was performed with the 7500 Real Time PCR System (Applied Biosystems). TGF-β1 mRNA expression was normalized to β-actin expression and was quantified using the relative standard curve method, followed by comparison with the results from control samples (unmanipulated B cells). For all reactions, each condition was performed in triplicate.

### Statistical analysis

Data are presented as mean ± SEM. For clinical scores, significance between groups was analyzed using the Mann-Whitney U test. A value of P < 0.05 was considered significant. All other statistical analysis was performed using a Student’s *t*-test or a two-way multiple-range analysis of variance test (ANOVA) for multiple comparisons. A value of p < 0.05 was considered significant.

## Additional Information

**How to cite this article**: Bjarnadóttir, K. *et al*. B cell-derived transforming growth factor-β1 expression limits the induction phase of autoimmune neuroinflammation. *Sci. Rep.* **6**, 34594; doi: 10.1038/srep34594 (2016).

## Supplementary Material

Supplementary Information
