# Supplementary material for: B cell-derived transforming growth factor-β1 expression limits the induction phase of autoimmune neuroinflammation
Source: Sci Rep. 2016 Oct 6;6:34594. doi: 10.1038/srep34594 (PMC5052622; doi:10.1038/srep34594)
Supplement: Supplementary Information [file srep34594-s1.pdf]

Supporting Information

B cell-derived transforming growth factor-β1 expression limits the induction phase of autoimmune neuroinflammation

Kristbjörg Bjarnadóttir[1], Mahdia Benkhoucha[1], Doron Merkler[1,2], Martin S. Weber[3,4], Natalie L. Payne[5], Claude C.A. Bernard[5], Nicolas Molnarfi[1,#] and Patrice H. Lalive[1,6,#,*]

[1]Department of Pathology and Immunology, School of Medicine, University of Geneva, Geneva, Switzerland.

[2]Division of Clinical Pathology, Geneva University Hospital, Geneva, Switzerland;

[3]Department of Neuropathology, University Medical Center, Georg August University, Göttingen, Germany.

[4]Department of Neurology, University Medical Center, Georg August University, Göttingen, Germany.

[5]Monash Regenerative Medicine Institute, Multiple Sclerosis Research Group, Monash University, Clayton, Victoria, Australia.

[6]Department of Neurosciences, Division of Neurology, University Hospital of Geneva and School of Medicine, Geneva, Switzerland.

[#]Co-senior author

Short title: B cell-derived TGF-β1 production in CNS autoimmunity

Key words: B cells, TGF-β1, regulation, EAE, multiple sclerosis

***Address correspondence to:** Prof. Patrice H. Lalive, Department of Neurosciences, Division of Neurology, University Hospital of Geneva and School of Medicine, Rue Gabrielle Perret-Gentil 4, 1211 Geneva 4, Switzerland, Phone: +41 22 372 83 18, Fax: +41 22 372 83 32, Email: patrice.lalive@hcuge.ch

Supplementary Figure S1

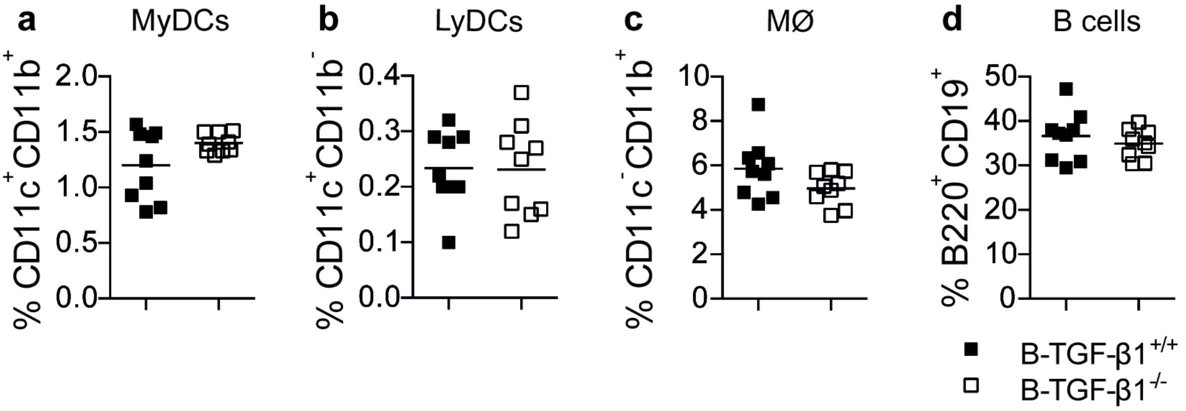

Supplementary Figure S1: Selective B cell TGF-β1−deficiency does not regulate the frequencies of APCs in naïve mice. APC subpopulations were characterized by flow cytometry from B–TGF-β1$^{+/+}$ (black) and B–TGF-β1$^{-/-}$ (open) naïve mice. Frequency of (**a**) myeloid (CD11c^{+}CD11b^{+}) DCs, (**b**) lymphoid (CD11c^{+}CD11b^{-}) DCs, (**c**) monocytes/macrophages (CD11c^{-}CD11b^{+}), (**d**) and B cells (B220^{+}CD19^{+}). Data are a composite of two independent experiments with similar results.

Supplementary Figure 2

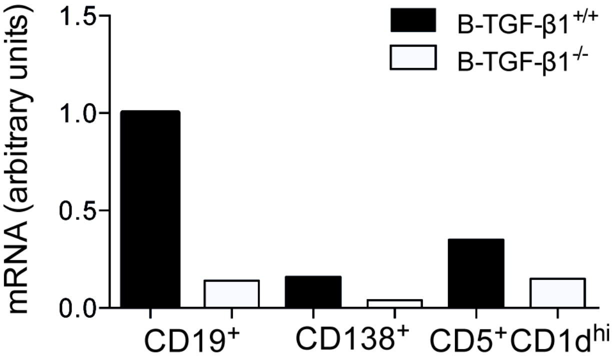

Supplementary Figure S2: Quantification of TGF-β1 expression by $CD19^{+}CD5^{-}$ $CD1d^{lo}$ B cells, $CD138^{+}$ plasma cells and $CD19^{+}CD5^{+}CD1d^{hi}$ B cells from B–TGF-$\beta1^{+/+}$ and B–TGF-$\beta1^{-/-}$ EAE mice. Levels of TGF-β1 mRNA in $CD19^{+}CD5^{-}$ $CD1d^{lo}$ B cells, $CD138^{+}$ plasma cells, and $CD19^{+}CD5^{+}CD1d^{hi}$ B cells (n=1) sorted from spleen of rmMOG-immunized B–TGF-$\beta1^{+/+}$ (black) and B–TGF-$\beta1^{-/-}$ (open) mice were assessed by quantitative real-time PCR. Data are from one experiment representative of two independent experiments with similar results.
